# Diabetes mellitus affects the treatment outcomes of drug-resistant tuberculosis: a systematic review and meta-analysis

**DOI:** 10.1186/s12879-023-08765-0

**Published:** 2023-11-20

**Authors:** Guisheng Xu, Xiaojiang Hu, Yanshu Lian, Xiuting Li

**Affiliations:** 1 Department of Preventive Medicine, Public Health Administration College, Jiangsu Health Vocational College, 69 Huang-shanling Road, Pukou District, Nanjing, Jiangsu Province 211800 China; 2 Department of Hygiene, Luhe District Center for Disease Control and Prevention, 8 Meteorological Road, Luhe District, Nanjing, Jiangsu Province 211500 China; 3 Department of Health Management and Medical Nutrition, Public Health Administration College, Jiangsu Health Vocational College, 69 Huang-shanling Road, Pukou District, Nanjing, Jiangsu Province 211800 China

**Keywords:** Drug-resistant tuberculosis, Multidrug-resistant tuberculosis, Diabetes mellitus, Treatment outcomes

## Abstract

**Background:**

Both tuberculosis (TB) and diabetes mellitus (DM) are major public health problems threatening global health. TB patients with DM have a higher bacterial burden and affect the absorption and metabolism for anti-TB drugs. Drug-resistant TB (DR-TB) with DM make control TB more difficult.

**Methods:**

This study was completed in accordance with the Preferred Reporting Items for Systematic Reviews and Meta-analysis (PRISMA) guideline. We searched PubMed, Excerpta Medica Database (EMBASE), Web of Science, ScienceDirect and Cochrance Library for literature published in English until July 2022. Papers were limited to those reporting the association between DM and treatment outcomes among DR-TB and multidrug-resistant TB (MDR-TB) patients. The strength of association was presented as odds ratios (ORs) and their 95% confidence intervals (CIs) using the fixed-effects or random-effects models. This study was registered with PROSPERO, number CRD: 42,022,350,214.

**Results:**

A total of twenty-five studies involving 16,905 DR-TB participants were included in the meta-analysis, of which 10,124 (59.89%) participants were MDR-TB patients, and 1,952 (11.54%) had DM history. In DR-TB patients, the pooled OR was 1.56 (95% CI: 1.24–1.96) for unsuccessful outcomes, 0.64 (95% CI: 0.44–0.94) for cured treatment outcomes, 0.63 (95% CI: 0.46–0.86) for completed treatment outcomes, and 1.28 (95% CI: 1.03–1.58) for treatment failure. Among MDR-TB patients, the pooled OR was 1.57 (95% CI: 1.20–2.04) for unsuccessful treatment outcomes, 0.55 (95% CI: 0.35–0.87) for cured treatment outcomes, 0.66 (95% CI: 0.46–0.93) for treatment completed treatment outcomes and 1.37 (95% CI: 1.08–1.75) for treatment failure.

**Conclusion:**

DM is a risk factor for adverse outcomes of DR-TB or MDR-TB patients. Controlling hyperglycemia may contribute to the favorite prognosis of TB. Our findings support the importance for diagnosing DM in DR-TB /MDR-TB, and it is needed to control glucose and therapeutic monitoring during the treatment of DR-TB /MDR-TB patients.

**Supplementary Information:**

The online version contains supplementary material available at 10.1186/s12879-023-08765-0.

## Introduction

Tuberculosis (TB) is a major public health issue that threatens global health, which caused 1.3 million deaths in 2020. The burden of TB is further aggravated by the growing prevalence of acquired immunodeficiency syndrome (AIDS), diabetes mellitus (DM) and kidney disease [1–3], as they may contribute to the TB risk and affect treatment outcomes [4–6]. With the changes in people’s lifestyles, the global burden of DM is continuously increasing. It is estimated that 693 million people worldwide will suffer from DM by 2045 [7]. The epidemic of DM will further aggravate the burden of TB, especially in low- and middle-income countries, DM and impaired glucose regulation were risk factors for TB in South Africa, which ORs were 2.4 (95% CI: 1.3–4.3) and 2.3(95% CI: 1.6–3.3), TB-DM patients also had higher odds of death(OR = 2.86,95%CI:1.08–7.62) in Italy [8, 9].

Multidrug-resistant TB (MDR-TB) is at least resistant to isoniazid and rifampicin, which may result from primary infection and treatment. MDR-TB is a serious threat for global TB control, and there are about 500,000 new cases of MDR-TB in each year all around the word [10]. According to the World Health Organization (WHO) estimated, there were 157,903 multidrug -resistant (MDR) TB cases reported in 2020, nearly 69% of cases were not diagnosed and treated in time [11]. Drug-resistant TB (DR-TB) and MDR-TB make controlling TB more challenging [12]. Patients afflicted with both DR-TB and DM will face worse treatment outcomes [13, 14], Some studies had shown that DM patients have a large bacterial load, which results in longer time to culture conversion and lengthen treatment. DM also can affect the absorption and metabolism for anti-TB drugs [15]. However, there were few systematic analyses to clarify and quantify the association between DM and DR/MDR-TB outcomes. Given the increasing burden of TB among people with DM, we performed a meta-analysis to systematically assess the association between DM and the treatment outcomes of DR/MDR-TB.

## Materials and methods

### Search strategy and study selection

We completed the Preferred Reporting Items for Systematic Reviews and Meta-analysis (PRISMA) guideline for this study. This systematic review has been registered with the International Prospective Register of Systematic Reviews (PROSPERO) (https://www.crd.york.ac.uk/prospero/ ID = CRD42022350214; registration number: CRD42022350214). We conducted a systematic search of the electronic database, including PubMed, Excerpta Medica Database (EMBASE), Web of Science, ScienceDirect and Cochrance Library by July 2022. We used the following search terms: (“Tuberculosis” or “Tuberculosis’s” or “Multidrug” or “Drug-resistant tuberculosis” or “Drug resistant tuberculosis” or “Multidrug-resistant tuberculosis” or “Multidrug resistant tuberculosis”) AND (“Diabetes mellitus” or “Diabetes insipidus” or “Diabetes” or “Mellitus” ) AND (“Treatment(s) outcome(s)”or “Treatment(s)”or “Outcome(s)”). The EndNote X9.0 software was used to manage records, screen, and exclude duplicates.

The inclusion criteria were as follows: (1) The study was designed as a cohort, case-control or cross-sectional study;(2)We did not set any specific exclusion criteria for the type of diabetes in DR/MDR-TB patients; (3) TB cases could provide whether there was a history of DM; (4) TB cases were diagnosed as DR/MDR-TB; (5) Treatment outcomes of TB cases were recorded, and the exclusion criteria were as follows: (1) No DM patients were involved in the treatment; (2) Only TB treatment outcomes; (3) Reviews/meta-analysis; (4) Treatment outcomes information only included sputum culture and/or smear; (5) Did not have enough outcomes to extract the value; (6) Other reasons for exclusion.

### Data extraction

Two reviewers extracted data independently and subsequently met to resolve discrepancies. In case of continued disagreement, a third reviewer made the final disposition. We extracted data on demographic characteristics, study design, location of the population, number of participants in each study, drug-resistant type, type of DM, score of quality assessment, adjusted odds ratio (OR) and relevant covariates (Table 1).

**Table 1:** Characteristics of the studies included for meta-analysis

Author and year	Country	Population	Study age-group(years)	Sex ratio Male/ Female	Study type	Sample size	Unsuccessful Outcomes (DM+)	Unsuccessful Outcomes (DM-)	Successful Outcomes (DM+)	Successful Outcome (DM-)	The type of DR-TB	The type of DM	Score of quality assessment	Odds ratio (95%Confidence Interval)
July Mary Johnson (2022)	India (Asian)	In-patients and out-patients	> 18	330/132	Case-control study	462	93	336	5	28	DR-TB	DM	7	1.5(0.58,4.13)
Daniel Bekele Ketema (2019)	Ethiopia (Africa)	In-patients and out-patients	All	283/225	Cohort study	508	7	79	9	413	DR-TB	DM	6	4.07(1.47,11.24)
A. Latif (2018)	Pakistan (Asian)	Community population	≥ 15	2970/2841	Cross-sectional study	5811	171	1631	338	3671	DR-TB	Type 2 DM	9	1.14(0.94,1.38)
Li Shi (2021)	China (Asian)	Hospital patients	≥ 18	196/18	Case-control study	214	10	7	97	100	MDR-TB	Type 2 DM	7	1.47(0.54,4.03)
Khasan Safaev (2021)	Uzbekistan (Asian)	Community population	All	412/133	Cohort study	545	13	229	7	296	MDR-TB	DM	7	2.40(0.94,6.11)
Subhakar Kandi (2021)	India (Asian)	In-patients and out-patients	All	201/176	Case-control study	377	11	151	20	195	MDR-TB	DM	5	0.71(0.33,1.53)
Wang Jianjie (2019)*	China (Asian)	In-patients and out-patients	All	137/415	Cohort study	552	60	97	89	306	MDR-TB	DM	6	2.13(1.43,3.17)
A K JanmeJa (2018)	India (Asian)	Community population	12–71	154/77	Case-control study	231	17	92	9	113	MDR-TB	DM	6	2.32(0.99,5.45)
Tariq Mahmood (2018)	India (Asian)	Hospital patients	> 20	106/35	Cross-sectional study	141	9	71	4	57	MDR-TB	DM	6	1.81(0.53,6.17)
Muñoz-Torrico (2017)	Mexico (America)	In-patients	NM	NM	Case-control study	77	25	15	18	19	MDR-TB	DM	5	1.76(0.71,4.36)
Baodong Yuan (2017)	China (Asian)	Hospital patients	≥ 18	245/105	Case-control study	359	32	59	42	226	MDR-TB	Type 2 DM	7	2.92(1.70,5.02)
Mohsen A. Gadallah (2015)	Egypt (Africa)	In-patients	7–76	161/67	Cohort study	228	17	53	19	139	MDR-TB	DM	6	2.35(1.13,4.85)
N. Kwak(2015)	Korea (Asian)	Hospital patients	NM	69/54	Case-control study	123	0	20	10	93	MDR-TB	DM	7	0.22(0.01,3.86)
J.Peter Cegielsk (2015)	Estonia, Latvia, Philippines, Peru, Russia, South Africa, Korea, Taiwan and Thailand (Europe, Asian and African)	Community population	≥ 18	609/364	Cohort study	973	25	226	107	615	MDR-TB	DM	6	0.64(0.40,1.01)
Matthew J. Magee (2014)	Georgia (Asian)	Community population	≥18	1153/268	Cohort study	1421	36	666	36	683	MDR-TB	DM	6	1.03(0.64,1.65)
Young Ae Kang (2013)	Korea (Asian)	Hospital patients	13–89	1039/368	Case-control study	1407	153	617	86	551	MDR-TB	DM	7	1.59(1.19,2.12)
Ma Tarcela Gler (2013)	Philippines (Asian)	Hospital patients	≥ 18	271/168	Cohort study	439	34	95	83	227	MDR-TB	DM	6	0.98(0.61,1.56)
L F Anderson (2013)	England, Wales and Northern Ireland(Europe)	Hospital patients	All	NM	Cohort study	191	6	41	4	140	MDR-TB	DM	6	5.12(1.38,19.02)
Shenjie Tang (2013)	China (Asian)	In-patients and out-patients	14 ~ 88	395/191	Case-control study	586	65	281	15	225	MDR-TB	DM	6	3.47(1.93,6.25)
Ekaterina V. Kurbatova (2012)	Russia, Latvia, Estonia, Peru and Philippines (Europe and Asian)	Out-patients	All	NM	Case- control study	1401	23	395	45	938	MDR-TB	DM	7	1.21(0.72,2.03)
Medea Gegia (2012)	Georgian (Asian)	the National TB Reference Laboratory	16–81	271/109	Cohort study	380	16	163	19	182	MDR-TB	DM	6	0.94(0.47,1.89)
D.Bendayan (2010)*	Israel (Asian)	In-patients and out-patients	16–93	102/30	Case- control study	132	6	34	11	81	MDR-TB	DM	7	1.3(0.44,3.80)
D.S.Jeon (2008)	Korea (Asian)	Hospital patients	All	NM	Case- control study	142	17	97	3	25	MDR-TB	DM	7	1.46(0.40,5.38)
T Yoshiyama (2005)	Japan (Asian)	In-patients and out-patients	All	NM	Case-control study	74	7	5	14	48	MDR-TB	DM	6	4.80(1.32,17.49)
Vaira Leimane (2005)	Latvia (Europe)	Hospital Patients, prisoners patients, Community population	17–78	NM	Case-control study	131	3	21	6	101	MDR-TB	DM	7	2.40(0.56,10.39)

### Treatment outcomes definitions

Treatment outcomes were divided into six categories, namely cured, treatment completed, treatment failed, death, lost to follow-up, and not evaluated. Cured and completed treatment were considered successful, and the rest were deemed unsuccessful in accordance with the WHO guidelines [16] (Table 2).

**Table 2:** Definitions of treatment outcomes for drug-resistant tuberculosis patients [16]

Treatment outcome	Definition
Cured	Treatment completed as recommended by the national policy without evidence of failure AND three or more consecutive cultures taken at least 30 days apart are negative after the intensive phase.^a^
Treatment completed	Treatment completed as recommended by the national policy without evidence of failure BUT no record that three or more consecutive cultures taken at least 30 days apart are negative after the intensive phase.^a^
Treatment failed	Treatment terminated or need for permanent regimen change of at least two anti-TB drugs because of: -Lack of conversion^b^ by the end of the intensive phase^a^, or -Bacteriological reversion in the continuation phase after conversion to negative, or -Evidence of additional acquired resistance to fluoroquinolones or second-line injectable drugs, or -Adverse drug reactions (ADRs).
Died	A patient who dies for any reason during the course of treatment.
Lost to follow-up	A patient whose treatment was interrupted for 2 consecutive months or more.
Not evaluated	A patient for whom no treatment outcome is assigned. (This includes cases “transferred out” to another treatment unit and whose treatment outcome is unknown)
Treatment success	The sum of cured and treatment completed.

^a^ For Treatment failed, lack of conversion by the end of the intensive phase implies that the patient does not convert within the maximum duration of intensive phase applied by the programme. If no maximumduration is defined, an 8-month cut-off is proposed. For regimens without a clear distinction between intensive and continuation phases, a cut-off 8 months after the start of treatment is suggested to determine when the criteria for Cured, Treatment completed and Treatment failed start to apply

^b^ The terms “conversion” and “reversion” of culture as used here are defined as follows:Conversion (to negative): culture is considered to have converted to negative when two consecutive cultures, taken at least 30 days apart, are found to be negative. In such a case, the specimen collection date of the first negative culture is used as the date of conversion

Reversion (to positive): culture is considered to have reverted to positive when, after an initial conversion, two consecutive cultures, taken at least 30 days apart, are found to be positive. For the purpose of defining Treatment failed, reversion is considered only when it occurs in the continuation phase

### Quality assessment

The quality of the included studies was evaluated using a modified version of the Newcastle-Ottawa Scale for cohort and case-control studies [17]. Studies were classified as having low (≥ 7 stars), moderate (5–6 stars), and high risk of bias (≤ 4 stars) with an overall quality score of 9 stars [18] (Table 1). For cross-sectional studies, we assigned each item of the AHRQ checklist a score of 1 (answered “yes”) or 0 (answered “no” or “unclear”). The high, moderate, and low risk of bias were identified as having a score of 0–3, 4–7, and 8–11, respectively (Table 1).

### Statistical analysis

Data was extracted using the Excel 2019 software, and further analyzed by Stata/se17.0. Heterogeneity between studies was assessed using the I^2^ statistic described by Higgins et al [19]. The pooled effects were estimated with fixed or random effect models: I^2^ ≤ 50% and *P* > 0.10 representing insignificant heterogeneity, using fixed-effects models; I^2^ ≥ 50% and *P* < 0.10 representing significant heterogeneity, using random-effects models [20].

The pooled effects of DM on DR/MDR-TB treatment outcomes were described by forests plots, quantified by OR (besides case-control studies, cross-sectional studies and cohort studies were also estimated by OR) and the corresponding 95% confidence interval (CI). *P* < 0.05 was considered as statistically significant. The publication bias was assessed through funnel plot and Egger’s test. All analyses were performed using the STATA 17.0 software (Texas, USA).

## Results

### Study selection and characteristics

**Fig. 1:**
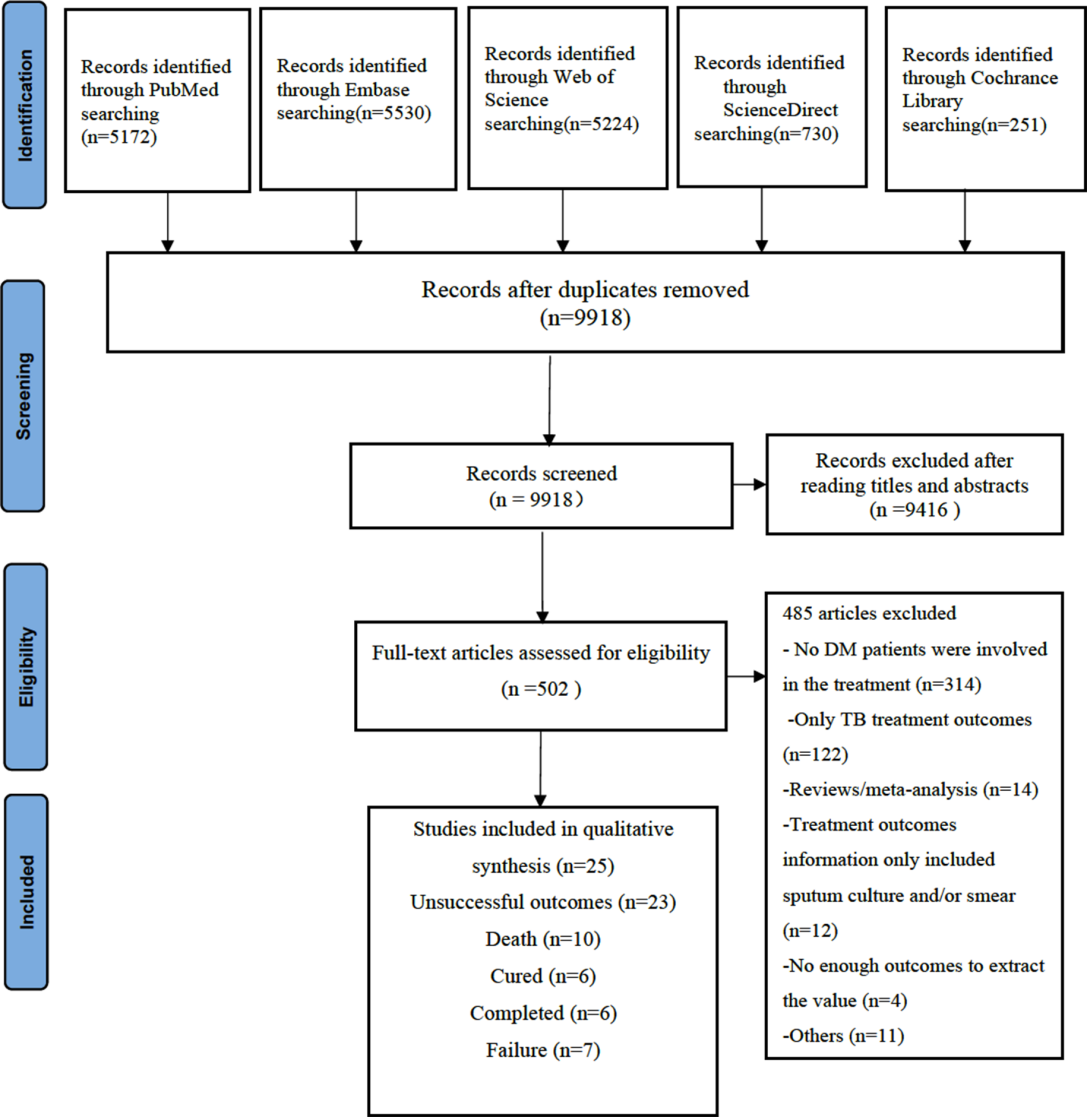
Flowchart of the study selection

We searched 9,918 papers by titles, abstracts and keywords and then excluded 9,416 papers without TB treatment outcomes. Among 502 articles under full-text reading, 477 articles were excluded for lacking targeted data or imperfect data (Fig. 1). Finally, we involved twenty-five eligible studies in the meta-analysis (Table 1) [13, 14, 21–43], including nine cohort studies, fourteen case-control studies and two cross-sectional studies. These studies were published from 2005 to 2022. Eleven studies were identified as having a low risk of bias, and fourteen studies had moderate risk of bias (Table 1). Twenty studies were conducted in Asian populations, four were in Europe populations, three were in African populations, and one was in American populations. The total sample size of subjects was 16,905 DR-TB patients, of which 10,124 (59.89%) participants were MDR and 1,952 (11.54%) had DM (DM+).

### Unsuccessful treatment outcomes

**Fig. 2:**
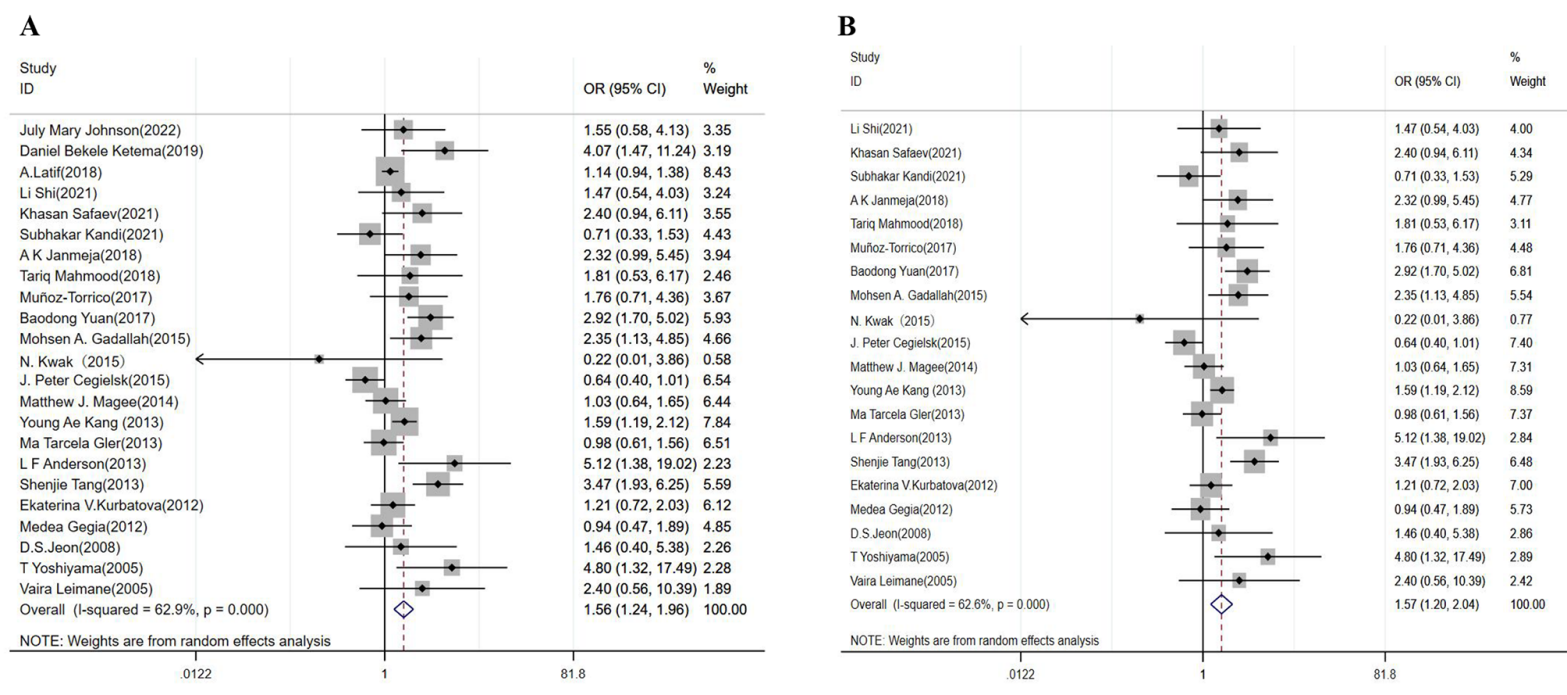
Forest plots for the association of diabetes mellitus with unsuccessful treatment outcomes for DR-TB (**A**) and MDR-TB (**B**)

**Fig. 3:**
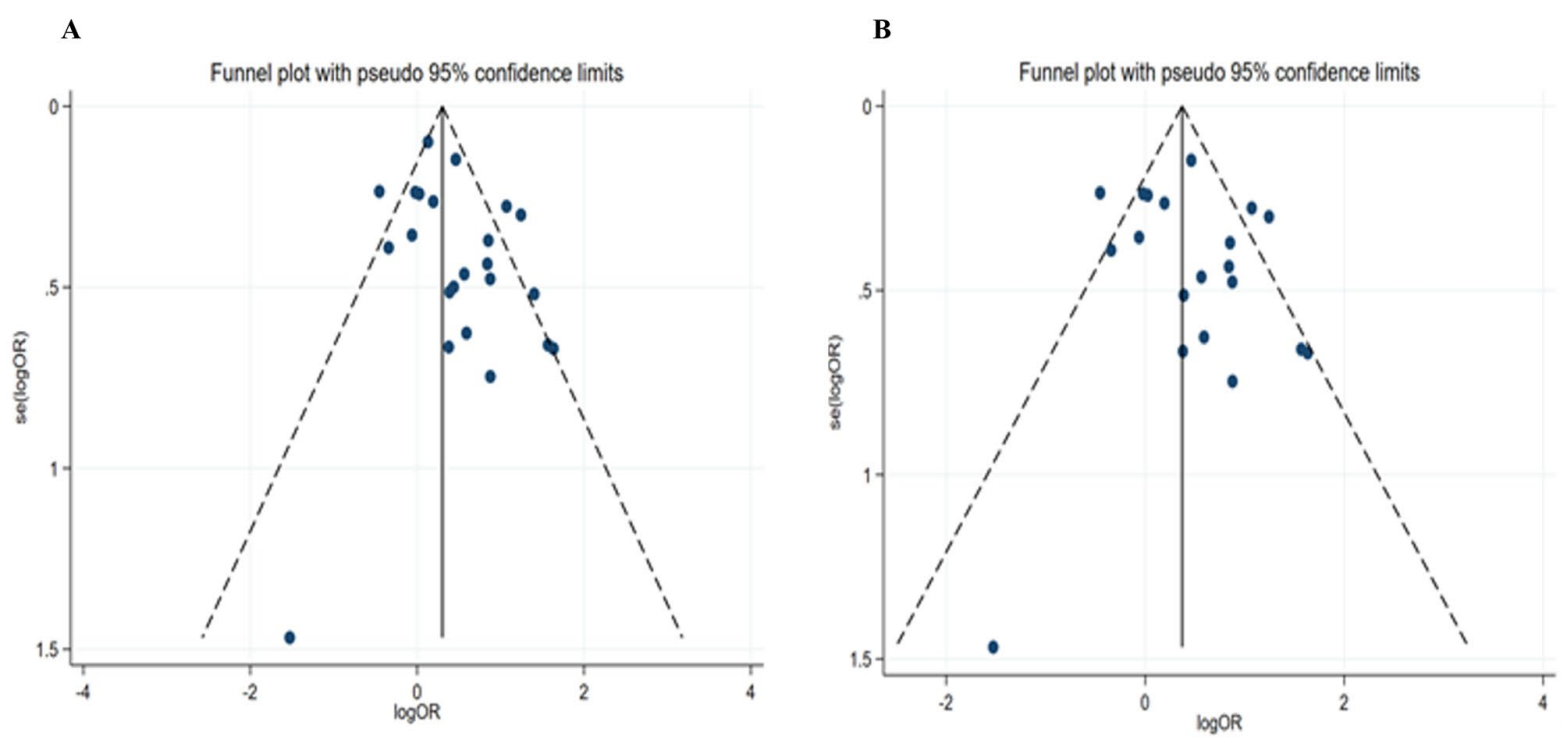
Funnel plot of the studies based on the association between DM and unsuccessful treatment outcomes for DR-TB (**A**) and MDR-TB (**B**)

Twenty-three studies analyzed the risk of DM on unsuccessful treatment outcomes in patients with DR-TB and twenty studies analyzed the risk of DM on unsuccessful treatment outcomes in patients with MDR-TB. DM patients were more likely to have unsuccessful treatment outcomes in DR-TB (OR = 1.56, 95% CI: 1.24–1.96) (Table 3; Fig. 2A) and MDR -TB patients (OR = 1.57, 95% CI: 1.20–2.04) (Table 3; Fig. 2B). Sensitivity analysis showed that four studies contributed the main heterogeneity [21, 27, 32, 35], which might be attributed to the inclusion of extensively drug-resistant (XDR-TB) [32, 35]. Figure 3 A and Fig. 3B illustrated the funnel plots of involved studies for DR- TB and MDR-TB patients with DM. We did not find the evidence for publication bias in DR-TB treatment outcomes (*P* = 0.086) and MDR-TB treatment outcomes (*P* = 0.365) by Egger’s test (Table 3).

**Table 3:** Pooled effects odds ratio (95% confidence interval), Heterogeneity test and Egger’s test for publication bias

DR-TB						MDR-TB				
Treatment outcomes	Odds ratio (95% CI)	I^2^ (%)	*P*-value for Heterogeneity	Z-value for Egger’s test	*P*- value for Egger’s test	Odds ratio (95% CI)	I^2^ (%)	*P*-value for Heterogeneity	Z-value for Egger’s test	*P*- value for Egger’s test
Unsuccessful outcomes	**1.56(1.24,1.96)**	62.9	< 0.001	1.35	0.086	**1.57(1.20,2.04)**	62.6	< 0.001	0.91	0.365
Death	1.32(0.97,1.82)	53.3	0.029	0.42	0.929	1.33(0.85,2.07)	59.2	0.016	0.25	0.940
Cured outcomes	**0.64(0.44,0.94)**	75.7	0.001	-1.69	0.062	**0.55(0.35,0.87)**	66.5	0.018	-0.98	0.263
Treatment completed outcomes	**0.63(0.46,0.86)**	0	0.660	0.98	0.221	**0.66(0.46,0.93)**	0	0.559	1.36	0.192
Treatment failed outcomes	**1.28(1.03,1.58)**	22.7	0.256	1.05	0.263	**1.37(1.08,1.75)**	19.7	0.284	0.94	0.275

### Death

**Fig. 4:**
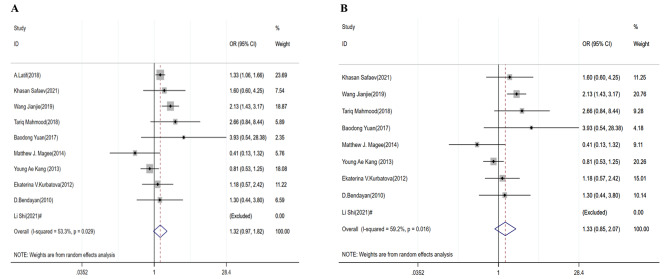
Forest plots for the association of diabetes mellitus with death treatment outcomes for DR-TB (**A**) and MDR-TB (**B**). # The number in this study was zero

We further compared the risk of death for DR/MDR-TB patients with and without DM. The random-effects model was used to estimate the pooled effects, as there was a significant heterogeneity for DR-TB studies (I^2^ = 53.3%, *P* = 0.029) and MDR-TB studies (I^2^ = 59.2%, *P* = 0.016) (Table 3). The pooled OR was 1.32 (95% CI: 0.97–1.82) and 1.33 (95% CI: 0.85–2.07), respectively (Table 3; Fig. 4A and B). There was no evidence for publication bias by Egger’s test (*P* = 0.929 in DR-TB; *P* = 0.940 in MDR-TB) (Table 3).

### Cured

**Fig. 5:**
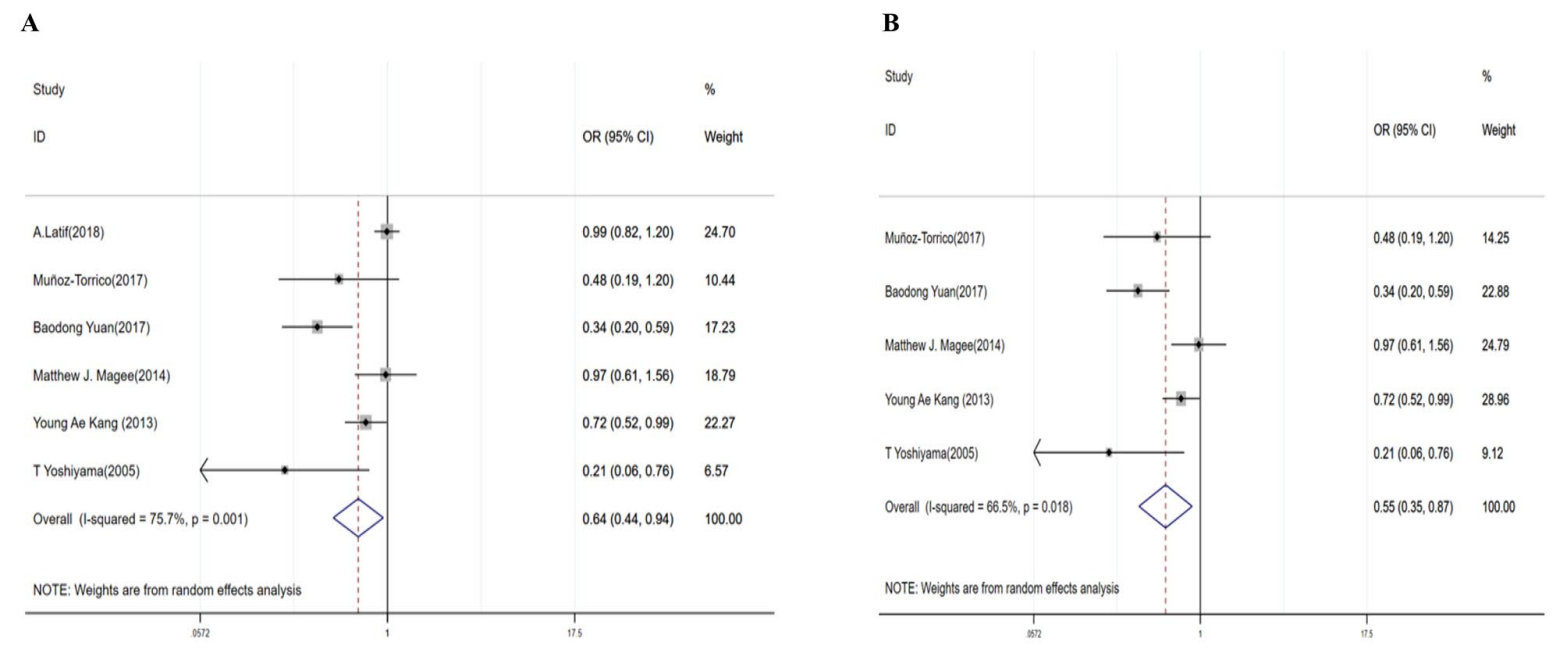
Forest plots for the association of diabetes mellitus with cured treatment outcomes for DR-TB (**A**) and MDR-TB (**B**)

DR/MDR-TB patients without DM were more likely to be cured (DR-TB: OR = 0.64, 95% CI: 0.44–0.94 (Table 3; Fig. 5A); MDR-TB: OR = 0.55, 95% CI: 0.35–0.87) (Table 3; Fig. 5B). The random-effects model was used as there was significant heterogeneity (DR-TB: I^2^ = 75.7%, *P* = 0.001; MDR-TB: I^2^ = 66.5%, *P* = 0.018). The Egger’s test suggested that there was no publication bias (*P* = 0.062 in DR-TB and *P* = 0.263 in MDR-TB).

### Treatment completed

**Fig. 6:**
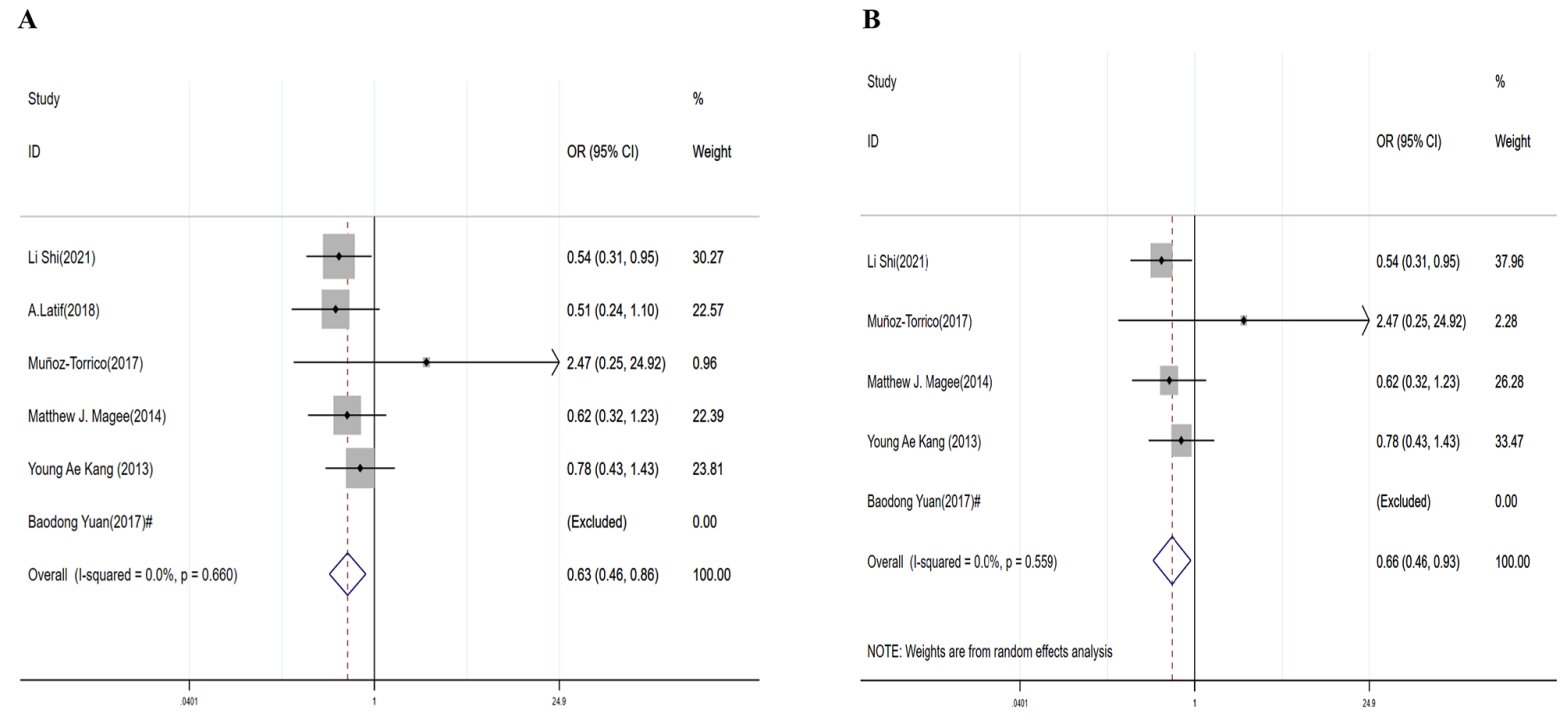
Forest plots for the association of diabetes mellitus with completed treatment outcomes for DR-TB(**A**) and MDR-TB(**B**). # The number in this study was zero

DR/MDR-TB patients without DM were more likely to complete treatment (DR-TB: OR = 0.63, 95% CI: 0.46–0.86 (Table 3; Fig. 6A); MDR-TB: OR = 0.66, 95% CI: 0.46–0.93) (Table 3; Fig. 6B). There was no evidence for heterogeneity (DR-TB: I^2^ = 0.00%, *P* = 0.660; MDR-TB: I^2^ = 0.00%, *P* = 0.559). There was no evidence for publication bias by Egger’s test (*P* = 0.221 in DR-TB and *P* = 0.192 in MDR-TB).

### Treatment failure

**Fig. 7:**
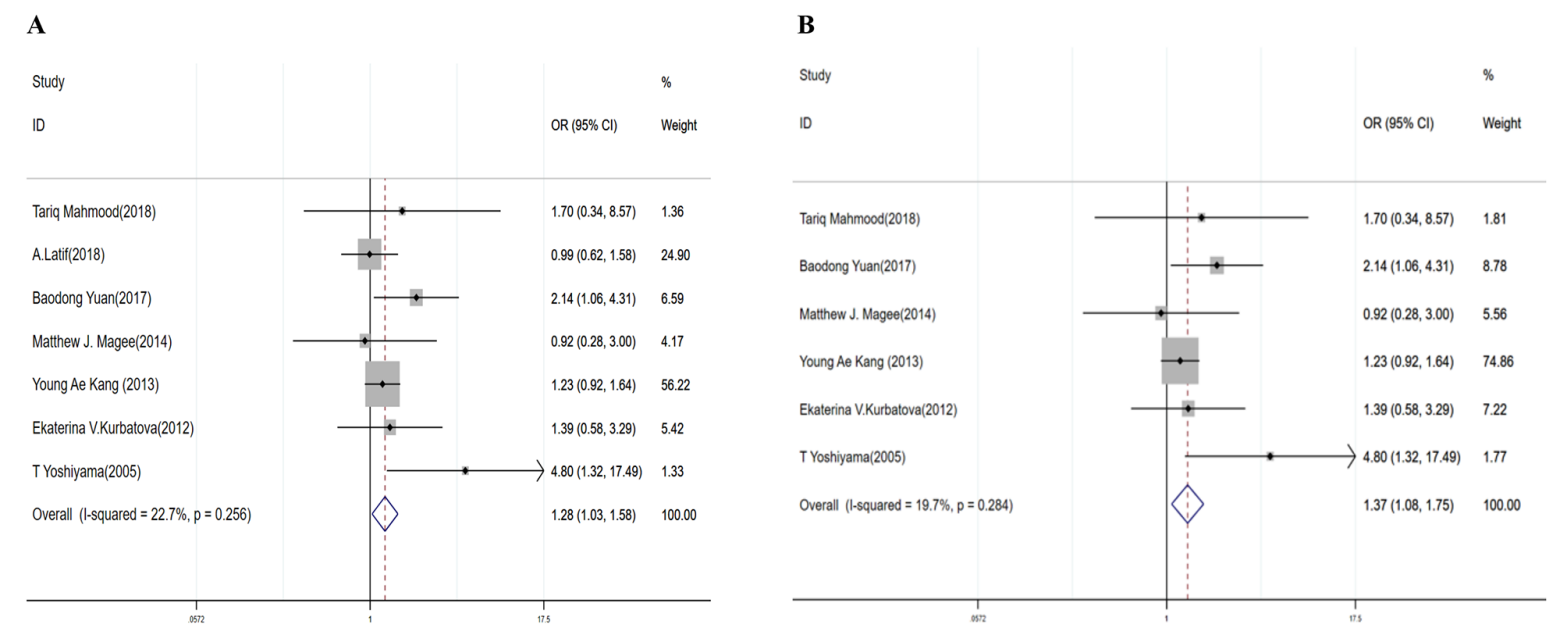
Forest plots for the association of diabetes mellitus with failed treatment outcomes for DR-TB (**A**) and MDR-TB (**B**)

DR/MDR-TB patients with DM were more likely to have treatment failed outcomes (DR-TB: OR = 1.28, 95% CI: 1.03–1.58 (Table 3; Fig. 7A); MDR-TB: OR = 1.37, 95% CI: 1.08–1.75) (Table 3; Fig. 7B). There was no evidence for heterogeneity (DR-TB: I^2^ = 22.7%, *P* = 0.256; MDR-TB: I^2^ = 19.7%, *P* = 0.284). There was no publication bias by Egger’s test (*P* = 0.263 in DR-TB and *P* = 0.275 in MDR-TB).

## Discussion

This study systematically reviewed the impact of DM on the treatment outcomes of DR/MDR-TB patients. We demonstrated the negative effect of DM on the prognosis of TB, which was consistent with the findings by Meghan and Sanju et al. [44, 45]. In this kinds topic research, previous systematic review and meta-analysis were focused on the treatment outcomes of TB and MDR-TB with DM, such as Huangfu and Tegegne et al. on treatment outcomes of TB and MDR-TB [9, 46]. Our study included treatment outcomes for both DR and MDR-TB patients with DM.

The prevalence of DM in TB patients was 11.54% (95% Cl: 11.06–11.93) in this study, which was lower than the global level (15.4%, 95% Cl: 14.1–16.6), and marginally higher as compared to the prevalence in Africa (9%, 95% Cl: 6.0–12.0) and China(7.8%, 95%CI:1.6–30.5)in Asian [47–49]. This result was most likely due to a higher proportion (88.0%) of African and Asian countries in our studies. The reason for this result is the difference of income in different countries and regions, for example, the study of Maier W al. show regional income plays a significant part in the explanation of diabetes prevalence [50].

DM can induce abnormalities in innate and adaptive immune responses, increasing the risk of the activation, complication, and outcomes of TB [51]. TB patients with DM have a rapidly progressive infection and a higher bacterial burden [52]. Coincident DM modulates Th1-, Th2-, and Th17-cell responses in latent TB in an IL-10- and TGF-β-dependent manner [53]. TB patients with DM had an increased risk of death and late culture transformation [54, 55].

The possible hypothesis of delay in the time of clearance and treatment failure of TB among DM patients is related to higher bacterial burden at diagnosis, which could be related to slower kinetics in the immune response in DM patients and altered pharmacokinetics of anti-TB drugs [55–58]. A pharmacokinetic study noted that plasma levels of rifampicin were 53% lower in TB patients with DM [59]. Depressed production of IFN-γ in DM patients is related to a decreased immune response to TB infection. The reduced IL-12 response to mycobacterial stimulation in leukocytes from TB with DM suggests a compromise of the innate immune response [60]. Roger et al. showed that TB patients with prediabetes or DM were more likely to have unsuccessful treatment outcomes in Peru, with an OR of 6.1 (95% Cl: 1.9–19.6) [61]. Siti et al. reported that TB patients with DM were three times more likely to have an unsuccessful treatment outcome than those without DM in Kelantan state, Malaysia [62]. MDR-TB is a type of TB, Therefore, the effect of glycemic control on treatment outcomes in TB patients with DM can also be applied to MDR-TB patients. Blood glucose control had a positive effect on the treatment outcome of TB patients with DM, An Indian study reported 30% fewer unsuccessful treatment outcomes (aOR = 0.72, 95% CI: 0.64–0.81) and 2.8 times higher odds of ‘no recurrence’ (aOR = 2.83, 95% CI: 2.60–2.92) among patients with optimal glycemic control at baseline [63]. Magee MJ et al. from Lima, Peru found reported faster culture conversion among those with glycemic control(aHR = 2.2,95% CI:1.1,4) [64]. There are some limitations to this study, Firstly, most of the included studies were from developing countries Asia and Africa and none were randomized controlled trials (RCT), which may have biased our research results. There were many factors that affected the severity of tuberculosis such as income level, temperature, and presence of other comorbidities. However, we found that a lot of relevant information could not be extracted in the original study, which may affect the generalization of finding.

In conclusion, DM is a risk factor for adverse outcomes in DR-TB or MDR-TB patients. Controlling hyperglycemia may contribute to a favorite prognosis of TB. Given the increasing burden of TB among people with DM, especially in areas with highly prevalent TB. It is needed to control glucose and therapeutic monitoring during the treatment of DR-TB /MDR-TB patients.

### Electronic supplementary material

Below is the link to the electronic supplementary material.

Supplementary Material 1

Supplementary Material 2

## Data Availability

The datasets used and/or analysed during the current study are available from the corresponding author on reasonable request.

## Abbreviations

TB: tuberculosis
DM: diabetes mellitus
DR-TB: Drug-resistant tuberculosis
MDR-TB: Multidrug-resistant tuberculosis
EMBASE: Excerpta Medica Database
ORs: odds ratios
CIs: confidence intervals
AIDS: acquired immunodeficiency syndrome
WHO: The World Health Organization
PRISMA: Preferred Reporting Items for Systematic Review and Meta-Analyses
PROSPERO: Prospective Register of Systematic Reviews
AHRQ: Healthcare Research and Quality
