# Supplementary material for: Diabetes mellitus affects the treatment outcomes of drug-resistant tuberculosis: a systematic review and meta-analysis
Source: BMC Infect Dis. 2023 Nov 20;23:813. doi: 10.1186/s12879-023-08765-0 (PMC10662654; doi:10.1186/s12879-023-08765-0)
Supplement: Supplementary file 1 — Supplementary Material 1 [file 12879_2023_8765_MOESM1_ESM.docx]

**Additional table: Search strategy in PubMed for TB/DR-TB/MDR-TB,**

**Diabetes mellitus , Treatment outcomes**

| Component | PubMed |
| --- | --- |
| Tuberculosis(#1) | "tuberculosi"[All Fields] OR "tuberculosis"[MeSH Terms] OR "tuberculosis"[All Fields] OR "tuberculoses"[All Fields] OR "tuberculosis's"[All Fields] |
| Drug-resistant tuberculosis(#2) | "tuberculosis, multidrug-resistant"[MeSH Terms] OR ("tuberculosis"[All Fields] AND "multidrug-resistant"[All Fields]) OR "multidrug-resistant tuberculosis"[All Fields] OR ("drug"[All Fields] AND "resistant"[All Fields] AND "tuberculosis"[All Fields]) OR "drug resistant tuberculosis"[All Fields] |
| Multi-drug resistant  Tuberculosis(#3) | "tuberculosis, multidrug-resistant"[MeSH Terms] OR ("tuberculosis"[All Fields] AND "multidrug-resistant"[All Fields]) OR "multidrug-resistant tuberculosis"[All Fields] OR ("multidrug"[All Fields] AND "resistant"[All Fields] AND "tuberculosis"[All Fields]) OR "multidrug resistant tuberculosis"[All Fields] |
| Diabetes mellitus(#4) | "diabete"[All Fields] OR "diabetes mellitus"[MeSH Terms] OR ("diabetes"[All Fields] AND "mellitus"[All Fields]) OR "diabetes mellitus"[All Fields] OR "diabetes"[All Fields] OR "diabetes insipidus"[MeSH Terms] OR ("diabetes"[All Fields] AND "insipidus"[All Fields]) OR "diabetes insipidus"[All Fields] OR "diabetic"[All Fields] OR "diabetics"[All Fields] OR "diabets"[All Fields] |
| Treatment outcome(#5) | "treatment outcome"[MeSH Terms] OR ("treatment"[All Fields] AND "outcome"[All Fields]) OR "treatment outcome"[All Fields] |
| Combined search | (#1OR #2 OR #3)AND #4 AND #5 |
