# Supplementary material for: Diabetes mellitus affects the treatment outcomes of drug-resistant tuberculosis: a systematic review and meta-analysis
Source: BMC Infect Dis. 2023 Nov 20;23:813. doi: 10.1186/s12879-023-08765-0 (PMC10662654; doi:10.1186/s12879-023-08765-0)
Supplement: Supplementary file 2 — Supplementary Material 2 [file 12879_2023_8765_MOESM2_ESM.docx]

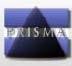
**PRISMA 2020 Checklist**

| **Section and Topic** | **Item #** | **Checklist item** | **Location where item is reported** |
| --- | --- | --- | --- |
| **TITLE** | | |  |
| Title | 1 | This report is a meta-analysis and systematic review. |  |
| ABSTRACT | | |  |
| Abstract | 2 | Background：Both tuberculosis (TB) and diabetes mellitus (DM) are major public health problems threatening global health. TB patients with DM have a higher bacterial burden and affect the absorption and metabolism for anti-TB drugs. Drug-resistant TB (DR-TB) with DM make control TB more difficult.  Purpose：We performed a meta-analysis to systematically assess the association between DM and the treatment outcomes of DR/MDR-TB, to provide a scientific basis for the prevention and control of DR-TB and MDR-TB patients with DM.  Methods: This study was completed in accordance with the Preferred Reporting Items for Systematic Reviews and Meta-analysis (PRISMA) guideline. This study was registered with PROSPERO, number CRD: 42022350214. We searched PubMed, Excerpta Medica Database (EMBASE), Web of Science, ScienceDirect and Cochrance Library for literature published in English until July 2022.  Study selection：Papers were limited to those reporting the association between DM and treatment outcomes among DR-TB and multidrug-resistant TB (MDR-TB) patients. Study methods included cohort study, case-control study, and cross-sectional study.  Limitations：The study included a small number of literatures on treatment outcomes for drug-resistant TB. Many may influence treatment outcomes factors unable to be extracted in the original study.  Conclusion: DM is a risk factor for adverse outcomes of DR-TB or MDR-TB patients. Controlling hyperglycaemia may contribute to the favourable prognosis of TB. DM diagnosis is needed before treatment for DR-TB and MDR-TB, and it is needed to control glucose and therapeutic monitoring during the treatment of DR-TB and MDR-TB patients. |  |
| INTRODUCTION | | |  |
| Rationale | 3 | Both TB and DM are major public health problems threatening global health. Clarify and quantify the association between DM and DR/MDR-TB outcomes are very import for TB control. There were few systematic analyses to clarify and quantify the association between DM and DR/MDR-TB outcomes. |  |
| Objectives | 4 | We performed a meta-analysis to systematically assess the association between DM and the treatment outcomes of DR/MDR-TB. To quantitatively analyse the effect of diabetes on the treatment outcomes of DR/MDR-TB. To provide a scientific basis for the prevention and control of DR-TB /MDR-TB patients with diabetes. |  |
| METHODS | | |  |
| Eligibility criteria | 5 | Papers were limited to those reporting the association between DM and treatment outcomes among DR-TB /MDR-TB patients, and corresponding treatment outcome data were available. |  |
| Information sources | 6 | We searched PubMed, Excerpta Medica Database (EMBASE), Web of Science, ScienceDirect, and Cochrance Library for literature published in English until July 2022. |  |
| Search strategy | 7 | We used the following search terms: (“Tuberculosis” or “Drug-resistant tuberculosis” or “Multidrug-resistant tuberculosis”) AND (“diabetes mellitus” or “diabetes”) AND (“Treatment(s) outcome(s)” or “treatment(s)”). Search strategy in additional table for PubMed database. |  |
| Selection process | 8 | The inclusion criteria were as follows: (1) The study was designed as a cohort, case-control, or cross-sectional study; (2) TB cases could provide whether there was a history of DM; (3) TB cases were diagnosed as DR/MDR-TB; (4) Treatment outcomes of TB cases were recorded. We searched 9,918 papers by titles, abstracts and keywords and then excluded 9,416 papers without TB treatment outcomes. Among 502 articles under full-text reading, 477 articles were excluded for lacking targeted data or imperfect data, Finally, we involved twenty-five eligible studies in the meta-analysis. Guisheng Xu and Xiaojiang Hu participated in literature search and review, The EndNote X9.0 software was used to manage records, screen, and exclude duplicates. |  |
| Data collection process | 9 | Data was extracted using the Excel 2019 software, two reviewers collected data from each report, and they worked independently, A total of twenty-five studies involving 16,905 DR-TB patients were included in the meta-analysis, of which 10,124 (59.88%) participants were MDR-TB patients, and 1,952 (11.54%) had DM history. Detailed data were showed in Table 1 of the manuscript. |  |
| Data items | 10a | We extracted data on demographic characteristics, study design, location of the population, number of participants in each study, drug-resistant type, and type of DM in table1.Treatment outcomes were divided into six categories, namely cured, treatment completed, treatment failed, death, lost to follow-up, and not evaluated. Cured and completed treatment were considered successful, and the rest were deemed unsuccessful in accordance with the WHO guidelines in table2. |  |
|  | 10b | Treatment outcome variables include cured，treatment completed, treatment failed, death, lost to follow-up, and not evaluated, cured, and completed treatment were considered successful, and the rest were deemed unsuccessful, Detailed denouement definitions were showed in table3 of the manuscript. The literature without full text was asked for help through the literature help function of the database. |  |
| Study risk of bias assessment | 11 | Publication bias was estimated by funnel plot, and Egger’s test in figure7 and table3. |  |
| Effect measures | 12 | The pooled effects of DM on DR/MDR-TB treatment outcomes were described by forest plots, quantified by OR (besides case-control studies, cross-sectional studies and cohort studies were also estimated by OR) and the corresponding 95% confidence interval (CI), Detailed data were showed in the manuscript (table1 and figure 2-6). |  |
| Synthesis methods | 13a | Papers were limited to those reporting the association between DM and treatment outcomes among DR-TB and MDR-TB patients. |  |
|  | 13b | Data was extracted using the Excel 2019 software, and further analysed by Stata/se17.0. Heterogeneity between studies was assessed using the I^2^ statistic described by Higgins et al. The pooled effects were estimated with fixed or random effect models: I^2^ ≤50% and P >0.10 representing insignificant heterogeneity, using fixed-effects models; I^2^ ≥50% and P <0.10 representing significant heterogeneity, using random-effects models. Literature with missing data was excluded. |  |
|  | 13c | The strength of association was presented as odds ratios (ORs) and their 95% confidence intervals (CIs) using the fixed-effects or random-effects models in each study, Detailed data were showed in figures2-6 of the manuscript. |  |
|  | 13d | The pooled effects were estimated with fixed or random effect models: I^2^ ≤50% and P >0.10 representing insignificant heterogeneity, using fixed-effects models; I^2^ ≥50% and P <0.10 representing significant heterogeneity, using random-effects models. Odds ratios (ORs) ,95% confidence intervals (CIs) and test of heterogeneity were analysed by Stata/se17.0. |  |
|  | 13e | Subgroup analysis was performed by analysing type of diabetes, location, and type of drug resistance (extensively resistant or not). |  |
|  | 13f | Sensitivity analysis was performed by stata17, the sensitivity was analyzed by means of literature exclusion. |  |
| Reporting bias assessment | 14 | The publication bias was assessed through a funnel plot and Egger’s test. All analyses were performed using the STATA 17.0 software (Texas, USA). |  |
| Certainty assessment | 15 | The treatment outcomes were determined by WHO guidelines, Literature quality was assessment by the Newcastle-Ottawa Scale and AHRQ checklist in table1. |  |

| Section and Topic | Item # | Checklist item | Location where item is reported |
| --- | --- | --- | --- |
| RESULTS | |  |  |
| Study selection | 16a | We searched 9,918 papers by titles, abstracts and keywords and then excluded 9,416 papers without TB treatment outcomes. Among 502 articles under full-text reading, 477 articles were excluded for lacking targeted data or imperfect data. The detailed description was in figure1 of the manuscript. |  |
|  | 16b | 477 articles excluded.1）No DM patients were involved in the treatment (n =314).2) TB treatment outcomes (n =122).3) Reviews/meta-analysis (n =14).4) Treatment outcomes information only included sputum culture and/or smear (n =12).5) Did not have enough outcomes to extract the value (n =4).6) Others (n =11). |  |
| Study characteristics | 17 | The specific characteristics of each paper were showed in the manuscript (table1 ). |  |
| Risk of bias in studies | 18 | Publication bias was analyzed by Heterogeneity test and Egger's test. |  |
| Results of individual studies | 19 | The pooled OR and 95% confidence intervals for corresponding treatment outcomes were showed in the manuscript (table 3 and figure 2-6). |  |
| Results of syntheses | 20a | The contribution of each study to the pooled OR results was shown in the manuscript (figure 2-6), The contribution of each study to the publication bias was shown in the manuscript (figure 7). |  |
|  | 20b | A total of twenty-five studies involving 16,905 DR-TB patients were included in the meta-analysis, of which 10,124 (59.88%) participants were MDR-TB patients, and 1,952 (11.54%) had DM history. In DR-TB patients, the pooled OR was 1.56 (95% Cl: 1.24-1.96) for unsuccessful outcomes, 0.64 (95% Cl: 0.44-0.94) for cured treatment outcomes, 0.63 (95% Cl: 0.46-0.86) for completed treatment, and 1.28 (95% Cl: 1.03-1.58) for treatment failure. Among MDR-TB patients, the pooled OR was 1.57 (95% Cl: 1.20-2.04) for unsuccessful treatment outcomes, 0.55 (95% Cl: 0.35-0.87) for cured treatment outcomes, 0.66 (95% Cl: 0.46-0.93) for completed treatment and 1.37 (95% Cl: 1.08-1.75) for treatment failure. The detailed results were in the manuscript (table3 and figures2-6). |  |
|  | 20c | There were differences in the type of diabetes patients, type of drug resistance, and regional differences in the included literature. Extensive drug resistance had a greater impact on heterogeneity. |  |
|  | 20d | The sensitivity analysis of all literatures showed that the type of multidrug resistance had a great influence on the overall sensitivity analysis. |  |
| Reporting biases | 21 | Seeing the funnel plot in the manuscript for details （figure7）and Egger’s test(table3). |  |
| Certainty of evidence | 22 | To quantitatively estimate the impact of diabetes on treatment outcomes in DR/MDR-TB.  the pooled OR for each treatment outcome were showed in the table in the manuscript (table 3). |  |
| DISCUSSION | |  |  |
| Discussion | 23a | This study systematically reviewed the impact of DM on the treatment outcomes of DR/MDR-TB patients. We demonstrated the negative effect of DM on the prognosis of TB, which was consistent with the findings by Meghan and Sanju et al. |  |
|  | 23b | Some treatment outcomes were not found in all studies. Such as lost to follow-up and not evaluated. |  |
|  | 23c | These results may not apply to TB or XDR-TB populations. |  |
|  | 23d | Controlling hyperglycaemia may contribute to the favourable prognosis of TB. Multi-centre and large sample population will be needed for verification in the future. |  |
| OTHER INFORMATION | |  |  |
| Registration and protocol | 24a | This systematic review has been registered with the International Prospective Register of Systematic Reviews (PROSPERO) (https://www.crd.york.ac.uk/prospero/ ID=CRD42022350214; registration number: CRD42022350214). |  |
|  | 24b | Can be available from: https://www.crd.york.ac.uk/prospero. |  |
|  | 24c | The article title had been revised, And added Xiuting Li as an author. |  |
| Support | 25 | This work was supported by Scientific Research Project of Jiangsu Health Vocational College-General Project (JKC201940), Project Leader: Guisheng Xu, Main Participants： Yansu Lian.Jiangsu University Philosophy and Social Science Research Project-General project-t(2020SJA0844), Project Leader: Guisheng Xu, Main Participants： Yansu Lian, Xiaojiang Hu.  Scientific Research Project of Jiangsu Provincial Health Commission (Z2019009), Project Leader: Xiuting Li. |  |
| Competing  interests | 26 | The authors declare that there is no conflict of interest. |  |
| Availability of data, code, and other materials | 27 | The information available includes tables1-3 and figures 1-7 in the manuscript. |  |

*From:*  Page MJ, McKenzie JE, Bossuyt PM, Boutron I, Hoffmann TC, Mulrow CD, et al. The PRISMA 2020 statement: an updated guideline for reporting systematic reviews. BMJ 2021;372:n71. doi:

10.1136/bmj.n71

For more information, visit:<http://www.prisma-statement.org/>
